# Functional Mapping of Dynamic Traits with Robust *t*-Distribution

**DOI:** 10.1371/journal.pone.0024902

**Published:** 2011-09-22

**Authors:** Cen Wu, Gengxin Li, Jun Zhu, Yuehua Cui

**Affiliations:** 1 Department of Statistics and Probability, Michigan State University, East Lansing, Michigan, United States of America; 2 College of Agriculture and Biotechnology, Zhejiang University, Hangzhou, Zhejiang, People's Republic of China; George Mason University, United States of America

## Abstract

Functional mapping has been a powerful tool in mapping quantitative trait loci (QTL) underlying dynamic traits of agricultural or biomedical interest. In functional mapping, multivariate normality is often assumed for the underlying data distribution, partially due to the ease of parameter estimation. The normality assumption however could be easily violated in real applications due to various reasons such as heavy tails or extreme observations. Departure from normality has negative effect on testing power and inference for QTL identification. In this work, we relax the normality assumption and propose a robust multivariate 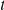-distribution mapping framework for QTL identification in functional mapping. Simulation studies show increased mapping power and precision with the 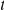 distribution than that of a normal distribution. The utility of the method is demonstrated through a real data analysis.

## Introduction

Since the seminal work of interval mapping [1], quantitative trait loci (QTL) mapping with molecular markers has been a standard means in targeting genetic regions harboring potential genes of interest underlying various traits of interest in biomedical and agricultural research. TL mapping originated for single trait analysis, then later was considered for multiple traits for the improvement of mapping precision and power (e.g., [2]). When a trait is measured through many developmental stages, e.g., body height measured over many time points, the trait reveals the dynamic expression of the underlying genes that are associated with the trait. These traits, which can be expressed as a function of time, were termed “function-valued traits” by Pletcher and Geyer [3] or “infinite-dimensional characters” by Kirkpatrick and Heckman [4]. Mapping QTLs or genes underlying the dynamics of a developmental characteristic has been a longstanding challenging topic in genetic mapping. Recently, Wu and his colleagues (e.g., [4]–[6]) have developed a series of mapping approaches for dynamic traits by integrating mathematical functions into a QTL mapping framework, opening a new era for genetic mapping. The so-called functional mapping approach enables one to propose either parametric or non-parametric functions to model the developmental mean function of a dynamic trait. By testing mean differences for different QTL genotype categories in a genome-wide linkage scan, one can identify potential genes that govern the dynamics of a trait.

In general, functional mapping assumes a joint multivariate normal distribution of a developmental trait. The mean of the multivariate normal is modeled through functions of time, and trait correlations among different developmental stages are fully considered. These treatments make functional mapping more powerful than single trait analysis for a developmental trait [4]. The multivariate normality assumption is commonly assumed for all the methods developed for functional mapping in the literature. In real data analysis, this assumption could be easily violated as in the case for single trait analysis [8]. In a single trait analysis, von Rohr and Hoeschele [8] showed that deviations from normality may lead to false positive QTL detection. The authors proposed to replace the normality assumption with the 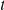-distribution to allow for heavy tails and skewness of a trait distribution. In human linkage analysis with the variance components model, Peng and Siegmund [9] also showed that departure from multivariate normality for the trait vector could dramatically reduce the mapping power when multivariate normality is assumed. As an alternative, the authors proposed to substitute the multivariate normal with a multivariate 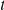-distribution and showed great power improvement.

For a developmental trait, the multivariate normality assumption is often a concern, especially for a small sample size. For many applied problems, the tails of the data distribution are often longer than a normal distribution assumes. In the presence of extreme observations, statistical inference based on the normal distribution is less robust. This could lead to low power or false positives under a functional mapping framework. The lack of robustness with respect to outliers and heavy tails that results from using a Gaussian model makes the multivariate 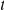-distribution a powerful alternative.

In this work, we relax the multivariate normality assumption in functional mapping and propose a robust multivariate 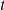-distribution for the error terms. The proposed method is implemented in a mapping framework that is different from Peng and Siegmund's treatment [9]. A mixture multivariate 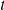-distribution is proposed and an expectation-maximization (EM) algorithm is derived to estimate various parameters of interests. To make the method more flexible for any developmental traits, a non-parametric B-spline technique is incorporated to model the developmental mean function. An antedependence covariance model is applied to model the non-stationary covariance structure [10]. Extensive simulations are conducted to evaluate the model performance. The utility of the method is demonstrated by reanalyzing a real data set for the purpose of identify genes underlying the variation of rice tiller numbers.

## Methods

### The mixture model and the multivariate 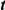 likelihood function

Consider a backcross design initiated with two inbreed lines with contrasting phenotypic difference. A genetic linkage map can be constructed with molecular markers. Suppose there is a putative segregating QTL, with alleles 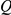 and 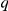, that affects the trait of interest, but by different degrees. For a backcross population with 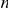 observations, each one is measured over 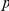 time points. The phenotypic vector 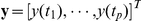 follows a multivariate distribution with a density function 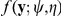, where 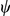 and 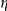 denote location and scale parameters.

In a QTL mapping study, the location and QTL genotype are generally unobservable. Suppose the QTL genotypes contributing to the variation of a dynamic quantitative trait are 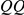 and 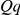. This missing data problem can be overcome by modeling the observed phenotypic data with a finite mixture model
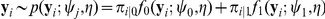
where 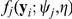 is the probability density function with the location parameters 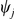 corresponding to QTL genotype 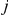 (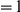 for 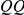 and 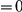 for 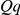); 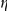 contains the scale parameters common to all components; and 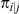 is the mixture proportion of individual 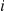 given the QTL genotype 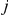. For a backcross design, the mixture proportions can be obtained via the conditional probabilities of QTL genotypes given the flanking marker in a standard backcross design [11].

As we mentioned in section Introduction, multivariate normality is a general concern in functional mapping when extreme observations or heavy tails are observed. To make the functional mapping more flexible, we assume the multivariate 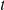 distribution for 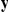. The multivariate 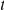 density function for individual 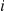 given genotype 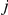 is given by
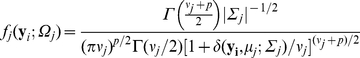
(1)where for genotype 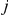 ( = 0, 1), 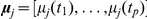 denotes the mean vector, 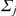 is a positive definite covariance matrix, 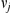 is the degree of freedom, and 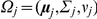 contains all the parameter of interest corresponding to genotype 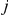. The Mahalanobis distance between 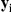 and 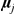 with respect to 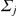 is denoted as
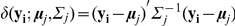



At a specific time point 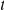, the relationship between the observation and the mean can be expressed by a linear model
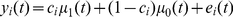
(2)where 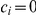 or 1 if the QTL genotype is 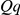 or 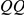, respectively; and 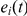 is the error term following a 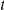 distribution with mean zero and variance 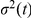. The errors at two different time points 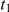 and 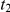, are correlated with correlation coefficient 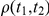.

Assuming independence among individuals, the joint likelihood function can be expressed as
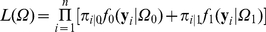
(3)where 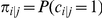, and 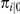
+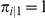. The unknown parameter vector 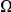 consists of two sets of parameters. One set, denoted as 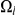, determines the locations of the QTL with respect to markers; and the other set, denoted as 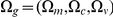, determines the multivariate 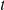 distribution of the trait corresponding to each QTL genotype, where 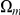, 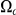 and 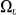 define the mean vectors, the covariance matrices and the degree of freedom.

### Modeling the dynamic mean function

One of the challenges in functional mapping lies in the complexity of the developmental pattern as well as the intra-individual variation of a longitudinal trait. Rather than estimating the discrete means at 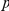 time points, functional mapping treats a developmental trait as a dynamic process which is fitted by a continuous function [7]. For a typical growth trait, a parametric logistic function would fit most data well [12] and it has been broadly applied in many applications (e.g., [5], [13]). For other developmental characteristics such as a process that experiences programmed cell death, it is infeasible to find a mathematic function to describe the process, thus a joint modeling approach may be an option (e.g., [14]). Legendre polynomials have been shown to be useful in modeling irregular developmental processes (e.g., [15], [16]). With recent statistical advances in nonparametric regression, a natural and flexible way to model an irregular developmental process is in a nonparametric fashion in which the data specify the best fit [17].

Here we adopt a nonparametric B-spline technique to model the time-dependent mean function. As aforementioned, the phenotype values are recorded at 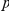 time points, denoted as 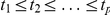. At a particular time point 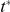, we can fit the dynamic genotypic means corresponding to the QTL genotypes 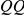 and 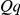 by using B-spline functions with different orders. Denote the B-spline basis function in a matrix as **B** which can be defined by the degree and the order of a piecewise polynomial. For the uniform quadratic B-spline with 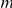
th order, the basis matrix is expressed as
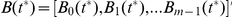
A column vector of the basis matrix 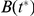 is called a base function. For the two QTL genotypes 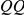 and 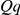 (corresponding to 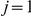 and 0 respectively), the base genotypic vector is expressed as 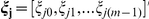. The vector contains the coefficients to be estimated for genotype 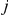. The B-spline function depends on the observed time points, the number and the relative positions of the knots. The criteria to determine the knots are open to discussion [17]. For the real data analyzed in this study, equidistantly distributed inner knots are selected since the rice tiller numbers are observed with the same duration (10 days). Around 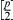 inner knots should be selected, as suggested in Yang et al [17]. We choose 3 evenly distributed knots and with this representation, the dynamic genotypic mean at time 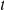, 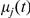, can be estimated by 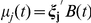. It is shown later on simulation study that the estimation on the mean curves is satisfactory. This serves as a credential for our choice. Further investigation also indicates that the estimation are not sensitive to various spline bases.

### Modeling the covariance function

Though nonparametric modeling of the time-dependent mean functions has been extensively studied, research on the modeling of the covariance structures via non-parametric approaches is rarely reported due to various difficulties [18]. In the original functional mapping [5], a stationary covariance function such as the first-order autoregressive (AR(1)) model was applied. Structured antedependence (SAD) model was later on adopted in functional mapping [19] for the purpose of relaxing the stationarity assumption. The SAD model is a non-stationary model which has been applied in many studies [20]. The SAD model with order 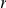 for modeling the error term in Eq. (2) is denoted by
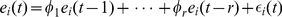
(4)where 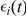 is the “innovation” term assumed to be independent and distributed as 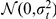; and 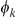 (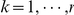) are the antedependence coefficients. Therefore, the variance-covariance matrix of the a developmental process can be expressed as
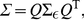
(5)where 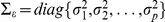 is a diagonal matrix. For the first-order SAD or SAD(1) model, the matrix **Q** can be expressed as
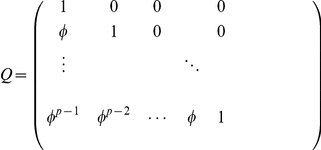
In general, the SAD order 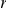 can be selected through an information criterion (see [19]). Since the purpose of this study is not to compare the performance of various modeling approaches for the covariance structure, we simply adopt the SAD(1) function due to its non-stationarity property and simplicity.

### Parameter estimation

The Expectation-Maximization (EM) algorithm, originally proposed by Dempster et al. [21], was applied to obtain the maximum likelihood estimates (MLEs) of the unknown parameters contained in 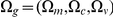. The detailed algorithm is given in the Appendix S1. Note that the QTL position is generally considered as an unknown parameter which can be estimated together with other mean and variance parameters. This, however, could dramatically increase the complexity of an estimation algorithm. As commonly treated in QTL mapping studies, we do not directly estimate the QTL-segregating parameters. Instead, we use a grid search approach to estimate the QTL location by searching for a putative QTL at every 1 or 2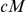 on an interval bracketed by two flanking markers. This linkage scan is done for the entire linkage map. The log-likelihood ratio test statistic for a QTL at a testing position is displayed graphically, to generate a log-likelihood ratio plot called the LR profile plot. The genomic position corresponding to a peak of the profile is the MLE of the QTL location.

### Hypothesis testing

Once the MLEs of parameters are obtained at each testing position, we are interested in testing whether there exists a QTL at a marker interval that governs the developmental process. The hypotheses for such a test can be formulated by
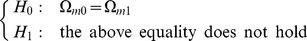
(6)The null hypothesis 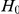 states that the data can be fitted by only one curve in the reduced model, while the alternative hypothesis 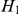 states that there exist two different curves to fit the data in the full model. The likelihood ratio test (LRT) has been the standard test in testing the QTL effect. Denote 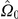 and 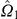 as the MLEs of the unknown poarameters under 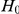 and 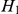, respectively. The LRT test statistic can be computed as the log-likelihood ratio of the reduced model to the full model, i.e., 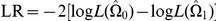. The genome-wide significance threshold can be determined through an empirical approach based on permutation tests proposed by Churchill and Doerge [22].

Following the overall genetic test described above, we can further test if a QTL triggers an effect on a certain time interval 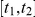 using a regional test approach based on the areas under the curve (AUC). The hypothesis for such a test can be formulated as
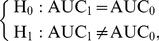
(7)where AUC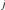 for genotype 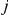 is calculated as 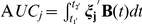. The significance of the test can be assessed through permutation tests [22].

## Results

### Simulation

We simulated a backcross population with a 100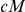 long linkage group, composed of 6 equidistant markers, under the assumption that QTL governs the whole developmental process. A putative QTL that affects a developmental process was assumed to be located 48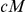 away from the first marker on the linkage group, in between the 3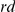 and 4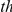 markers. The Haldane map function was used to convert the map distance into the recombination fraction. A developmental trait with 9 equally spaced time points was generated under various combinations of heritability levels (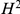 = 0.1 vs 0.4) and sample sizes (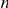 = 100 vs 400). The covariance was simulated assuming a first-order SAD structure.

In the simulation, we evaluated how well the parameters (including the QTL position as well as the mean and covariance parameters) can be estimated, how robust the multivariate 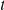 statistic is when data generate from a multivariate normal, and how poor the performance of multivariate mixture normal will be if the model is misspecified. Several simulation scenarios were considered. Tables 1 and 2 list the results assuming that the data generating and data analyzing models were the same. Tables 3 and 4 list the results assuming the data generating and analyzing models were not the same. In all simulation scenarios, we observed that increases in sample size and heritability always lead to more accurate parameter estimations. For example, in Table 1, the standard error for the mean parameter 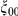 of genotype 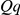 reduces from 0.14 to 0.06 while the sample size increases from 100 to 400 under a heritability level of 0.1. Meanwhile, given a sample size 400, the standard error decreases from 0.06 to 0.03 as H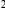 increases from 0.1 to 0.4, a two-fold decrease.

**Table 1:** The MLEs and standard errors (in the parenthesis) of the model parameters and the QTL position derived from 100 simulation replicates.

	H 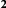 = 0.1		H 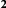 = 0.4	
True Parameters	n = 100	n = 400	n = 100	n = 400
QTL position				
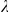 = 48	48.02(7.06)	48.14(2.19)	47.42(2.94)	47.6(1.53)
Mean Parameters for 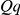				
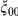 = 1.234	1.211(0.14)	1.214(0.06)	1.212(0.06)	1.209(0.03)
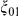 = 7.708	7.409(0.27)	7.364(0.15)	7.429(0.13)	7.452(0.08)
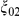 = 10.628	11.436(0.37)	11.433(0.20)	11.283(0.32)	11.248(0.16)
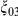 = 6.094	6.521(0.36)	6.461(0.22)	6.384(0.22)	6.397(0.11)
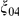 = 6.294	6.652(0.36)	6.600(0.18)	6.530(0.19)	6.531(0.09)
Mean Parameters for 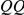				
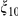 = 1.146	1.191(0.14)	1.173(0.07)	1.176(0.06)	1.165(0.03)
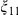 = 6.674	7.017(0.32)	6.989(0.14)	6.925(0.16)	6.929(0.07)
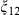 = 13.214	12.411(0.44)	12.419(0.20)	12.609(0.26)	12.564(0.13)
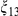 = 7.345	6.935(0.42)	6.965(0.17)	7.037(0.18)	7.026(0.10)
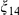 = 7.290	6.957(0.39)	6.998(0.17)	7.047(0.18)	7.036(0.10)
Covariance parameters				
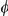 = 0.95	0.948(0.02)	0.948(0.01)	0.945(0.02)	0.946(0.01)
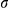	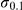 = 0.923		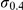 = 0.154	
	0.997(0.11)	1.007(0.04)	0.217(0.03)	0.220(0.01)
Degree of freedom				
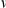 = 3	3.361(0.59)	3.203(0.25)	3.783(0.75)	3.786(0.42)

Data were simulated and analyzed with the proposed mixture multivariate 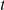 model (MVTT).

**Table 2:** The MLEs and standard errors (in the parenthesis) of the model parameters and the QTL position derived from 100 simulation replicates.

	H 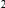 = 0.1		H 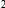 = 0.4	
True Parameters	n = 100	n = 400	n = 100	n = 400
QTL position				
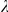 = 48	46.28(5.41)	48(1.75)	48.4(2.43)	48.04(1.36)
Mean Parameters for 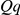				
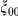 = 1.267	1.239(0.13)	1.225(0.07)	1.238(0.05)	1.23(0.03)
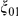 = 8.056	7.657(0.25)	7.667(0.12)	7.691(0.13)	7.67(0.06)
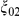 = 10.951	11.799(0.38)	11.782(0.17)	11.792(0.23)	11.825(0.11)
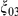 = 6.314	6.684(0.34)	6.700(0.19)	6.709(0.18)	6.722(0.07)
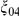 = 6.492	6.757(0.32)	6.785(0.19)	6.786(0.17)	6.796(0.07)
Mean Parameters for 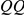				
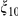 = 1.169	1.212(0.13)	1.193(0.07)	1.209(0.05)	1.198(0.03)
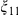 = 6.904	7.361(0.26)	7.279(0.13)	7.286(0.12)	7.269(0.06)
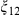 = 13.604	12.716(0.35)	12.733(0.17)	12.804(0.20)	12.766(0.10)
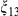 = 7.571	7.211(0.38)	7.180(0.18)	7.203(0.18)	7.181(0.08)
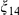 = 7.425	7.132(0.37)	7.146(0.16)	7.144(0.16)	7.133(0.08)
Covariance parameters				
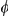 = 0.95	0.945(0.02)	0.946(0.01)	0.940(0.01)	0.939(0.01)
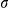	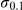 = 0.916		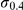 = 0.153	
	0.971(0.05)	0.983(0.02)	0.219(0.01)	0.222(0.01)

Data were simulated and analyzed with a mixture multivariate normal model (MVNN).

**Table 3:** The MLEs and standard errors (in the parenthesis) of the model parameters and the QTL position derived from 100 simulation replicates.

	H 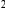 = 0.1		H 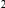 = 0.4	
True Parameters	n = 100	n = 400	n = 100	n = 400
QTL position				
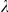 = 48	47.84(11.22)	47.16(3.83)	47.98(5.17)	48.12(1.55)
Mean Parameters for 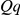				
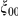 = 1.234	1.259(0.23)	1.227(0.12)	1.209(0.10)	1.213(0.04)
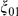 = 7.708	7.458(0.41)	7.343(0.21)	7.383(0.18)	7.370(0.09)
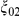 = 10.628	11.461(0.56)	11.466(0.29)	11.469(0.31)	11.470(0.14)
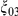 = 6.094	6.550(0.61)	6.481(0.29)	6.520(0.23)	6.517(0.14)
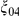 = 6.294	6.659(0.59)	6.593(0.27)	6.661(0.24)	6.625(0.13)
Mean Parameters for 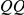				
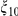 = 1.146	1.203(0.23)	1.196(0.12)	1.170(0.17)	1.175(0.04)
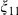 = 6.674	6.996(0.43)	7.017(0.22)	7.025(0.20)	7.011(0.09)
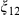 = 13.214	12.340(0.61)	12.397(0.30)	12.393(0.31)	12.369(0.14)
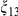 = 7.345	6.895(0.70)	6.930(0.33)	6.926(0.27)	6.907(0.14)
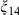 = 7.290	6.958(0.68)	6.954(0.32)	6.963(0.26)	6.942(0.13)
Covariance parameters				
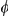 = 0.95	0.948(0.05)	0.946(0.03)	0.944(0.036)	0.945(0.02)
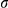	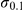 = 0.923		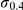 = 0.154	
	2.575(0.92)	2.662(0.48)	0.542(0.61)	0.520(0.10)

Data were simulated with the proposed mixture multivariate 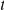 model, but analyzed with the mixture multivariate normal model (MVTN).

**Table 4:** The MLEs and standard errors (in the parenthesis) of the model parameters and the QTL position derived from 100 simulation replicates.

	H 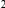 = 0.1			H 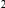 = 0.4	
True Parameters	n = 100	n = 400		n = 100	n = 400
QTL position					
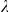 = 48	48.3(4.05)	48.24(1.93)		48.1(2.69)	47.9(1.34)
Mean Parameters for 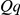					
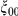 = 1.267	1.233(0.15)	1.248(0.07)		1.246(0.06)	1.236(0.03)
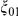 = 8.056	7.685(0.26)	7.704(0.13)		7.721(0.15)	7.712(0.08)
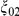 = 10.951	11.798(0.38)	11.799(0.17)		11.730(0.26)	11.740(0.14)
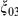 = 6.314	6.692(0.40)	6.731(0.18)		6.698(0.18)	6.685(0.09)
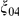 = 6.492	6.734(0.38)	6.800(0.17)		6.779(0.16)	6.766(0.08)
Mean Parameters for 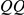					
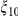 = 1.169	1.220(0.14)	1.193(0.07)		1.204(0.06)	1.196(0.03)
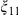 = 6.904	7.312(0.26)	7.255(0.13)		7.266(0.13)	7.254(0.07)
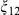 = 13.604	12.740(0.36)	12.737(0.18)		12.810(0.25)	12.796(0.13)
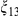 = 7.571	7.192(0.37)	7.157(0.16)		7.201(0.17)	7.193(0.09)
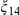 = 7.425	7.151(0.35)	7.120(0.15)		7.149(0.15)	7.142(0.09)
Covariance parameters					
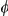 = 0.95	0.946(0.02)	0.947(0.01)		0.939(0.01)	0.940(0.01)
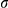	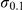 = 0.916			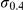 = 0.153	
	0.959(0.05)		0.969(0.02)	0.209(0.01)	0.212(0.01)
Degree of freedom					
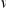	190.416(107.47)		206.02(97.05)	94.466(90.36)	75.988(70.40)

Data were simulated with a mixture multivariate normal model, but analyzed with the proposed mixture multivariate 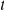 model (MVNT).

For a multivariate 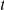 distribution, the degree of freedom (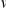) controls the shape of the distribution. A small value for 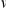 indicates that the normal assumption might be inappropriate for the data. Assuming 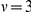, we simulated data assuming a multivariate 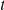 distribution. Table 1 (denoted as MVTT) shows that the parameters can be reasonably estimated with good precision. When both sample size and heritability level increase, the precision for the QTL position estimation is improved with reduced standard error. The same simulated data were further analyzed assuming a multivariate normal distribution for the error term. The results are tabulated in Table 3 (denoted as MVTN). It is clear that when the error distribution is misspecified, large standard errors were observed for all the parameters. In particular, the QTL position is poorly estimated with large standard errors under a small sample size and low heritability level. For example, the standard error increases from 2.94 to 5.17 under 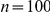 and 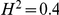, when data were analyzed with the proposed and the multivariate normal model. Under a small sample size, the multivariate 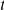 distribution is more robust than a multivariate normal.

Next we simulated data under the multivariate normal assumption and analyzed the data with the corresponding data generating model (denoted as MVNN) and the proposed 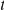 distribution model (denoted as MVNT). We used the results in Table 2 as a reference to compare the performance of the multivariate 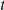 model in Table 4, since the results in Table 2 was obtained with the true model. Under a small sample size (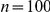) and low heritability level (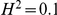), not surprisingly the results with the multivariate 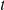 model are better for the multivariate normal model. For example, the standard error for the QTL position estimate is 4.35 in MVNT, while it is 5.41 in MVNN. Moreover, the bias in MVNN is also larger (1.72 vs 0.3). This result demonstrates the robustness of the 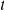 modeling under small samples. As sample size and heritability level increase, the results are very comparable. In real applications, due to various source of noise and for better estimation of the QTL position, a safe strategy is to apply the mixture multivariate 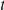 model in functional mapping.

**Figure 1:**
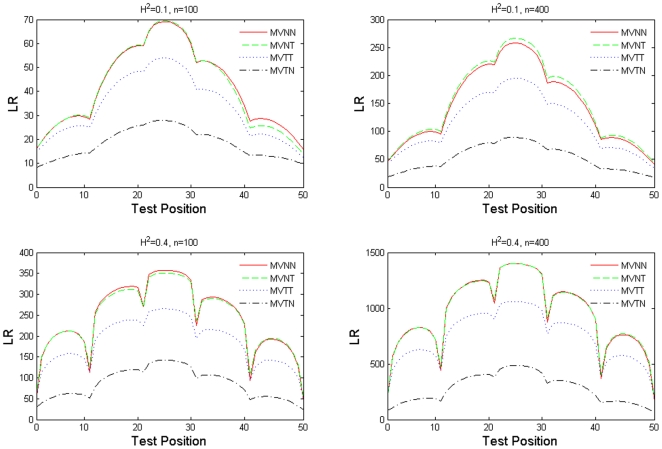
The LR profile plots averaged over 100 simulation replicates under different sample sizes (100 and 400) and heritability levels (0.1 and 0.4). The arrow sign indicates the simulated true QTL position.

In functional mapping, the likelihood ratio (LR) statistic is used as the indicator of a QTL signal. The larger the LR value at a genomic position, the stronger the evidence of a QTL at that position. The LR test statistics for the above four scenarios are also compared across the simulated genetic linkage group, averaged over 100 simulation replicates. Figure 1 explicitly displays the difference in LR values under the different combinations of sample size and heritability level. When data were generated assuming a multivariate normal distribution, the results obtained with the 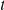 model (dashed curve) are very similar to those obtained with the normal model (solid curve). However, when data were generated assuming the multivariate 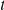 distribution, the 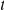 model (dotted curve) clearly outperforms the normal model (dash-dotted curve). This evidence indicates the superiority and robustness of the multivariate 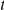 mixture model in functional mapping.

### A case study

We applied the method to a real data set to identify QTLs governing the variation of rice tiller number development to show the utility of the approach. A detailed description of the data can be found in Huang et al. [23] and Yan et al. [24]. In brief, semi-dwarf IR64 and tall Azucena, two inbred lines, were crossed to generate an F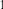 progeny population. A doubled haploid (DH) population of 123 lines was constructed through doubling haploid chromosomes of the F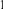 gametes. For this population, 40 isozyme and RAPD markers, and 135 RFLP markers were genotyped to construct a genetic linkage map of length 2005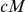 covering 12 rice chromosomes. Tiller numbers were measured every 10 days from 10 days after transplanting until all lines had headed. Nine developmental measurements were recorded for each rice. A plot of the original data can be found in Fig. 2 of Cui et al. [14].

**Figure 2:**
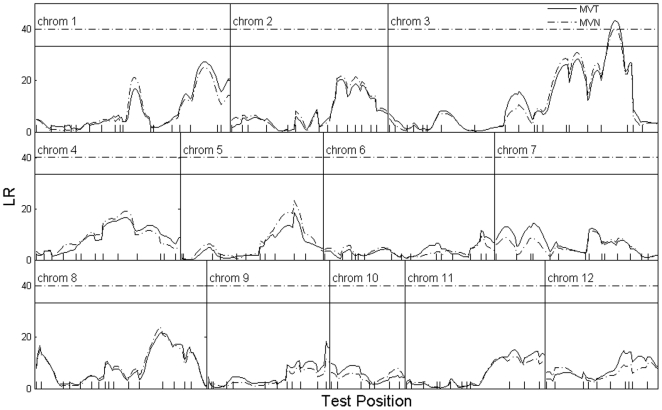
The LR profile plot across the 12 rice chromosomes, fitted with the proposed multivariate 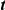
 **mixture model (solid curve) and a multivariate normal mixture model (dash-dotted curve).** The genomic position corresponding to the peak of the curve is the MLE of the QTL location (indicated by the arrows). The 5% genome-wide threshold value for claiming the existence of a QTL is given as the horizonal dotted and dash-dotted lines for the two models. The marker positions on the linkage groups are indicated as ticks [23].

We performed a genome-wide linkage scan at every 2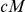 interval to locate potential QTLs that trigger effects for the programmed cell death of rice tillers. Figure 2 shows the genom-wide log-likelihood ratio profile plots, where the results obtained with the multivariate 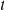 and the multivariate normal models are indicated by the solid and dashed curves, respectively, with the respective 5% genom-wide permutation threshold indicated by the horizontal solid and dashed lines (obtained with 1,000 permutations). The plot indicates one QTL located in chromosome 3 between marker 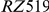 and 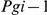. The QTL was also reported in our previous analysis [14], [15]. The other peaks did not pass the genome-wide significance threshold. A test of multivariate normality for the phenotype data without considering the marker data shows evidence of departure from normality, indicating that a multivariate 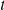 model may be more appropriate for the data. The LR values for the two models across the 12 chromosomes are very comparable, with the multivariate 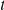 model generating slightly higher LR values in many positions.

**Figure 3:**
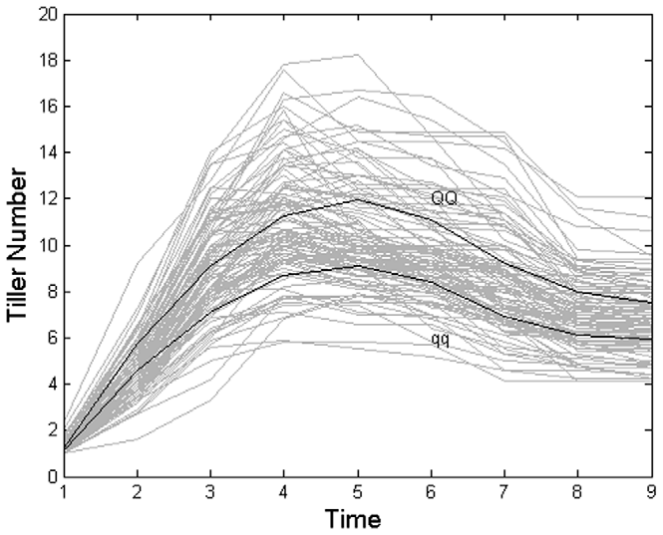
Two dynamic variation curves of tiller numbers corresponding to the two genotypes, 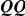
 **and** 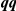 **.** All tiller number trajectories under study are shown in grey background.

The estimated QTL position on chromosome 3 and the corresponding marker interval as well as the MLEs of the model parameters are tabulated in Table 5. The tiller number developmental trajectories of the detected QTL are shown in Figure 3, with tiller number trajectories for all individuals indicated in the background. The gap between the two trajectories over the developmental stages is quite clear, indicating a developmental mean difference in tiller number between individuals carrying the two different genotypes. Individuals carrying genotype 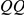 have high mean tiller numbers during the observed developmental stage, hence are preferable for selection in breeding.

**Table 5:** The QTL location and MLEs of the estimated parameters with the SAD(1) covariance structure.

QTL position	Marker Interval	Mean parameters for 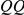				
( 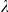 )		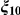	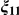	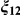	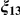	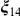
262 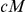	RZ519–Pgi-1	1.244	8.007	13.324	7.634	7.530

## Discussion

Functional mapping has been shown to be a powerful approach and also a standard means in mapping QTLs underlying the dynamics of quantitative traits [7]. However, most current methods in functional mapping assume a multivariate normal distribution for the time-course error term, which could be easily violated in reality. In this work, we extended the current functional mapping approach assuming a robust multivariate 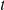 distribution for the error term, built upon the maximum likelihood framework while implemented with a full EM algorithm to estimate the model parameters. Extensive simulations show that the proposed model outperforms the mixture multivariate normal model when the underlying distribution is from a multivariate 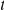 distribution. Even if the underlying distribution is normal, the proposed 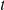 modeling approach performs as well or even better than the normal model (especially under a small sample size). Given its robustness, the proposed 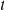 model should be adopted in a regular functional mapping study, especially when the sample size is small.

In the original functional mapping study, a developmental mean process is generally modeled with a mathematical function such as the logistic function for a growth trait [5]. In this study, we modeled the developmental mean process using a nonparametric spline technique, given its flexibility in modeling patterns of data distribution which does not follow any particular mathematical form (e.g., [17], [25]). The correlation structure was modeled by the non-stationary SAD model, which was studied in Zhao et al. [19] for functional mapping. Since the focus of this work is not on the modeling of the mean and the correlation structure, we simply adopted these approaches and did not compare the impact of different modeling approaches on the power of QTL identification. This investigation will be considered in our future work.

In real data analysis, there is not much significant deviation between the LR profile plot of the mixture 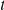 and the normal model. This is due to the fact that the data distribution is quite close to the multivariate normal. The same data were analyzed before with different models to approximate the developmental mean process [14], [15]. The QTL showing genome-wide significance in this study is consistent with the one found in our previous work, while some other QTLs in chromosome 1 reported in Cui et al. [15] did not pass genome-wide significance in this analysis. This is largely due to differences in the modeling of the mean process. As previous investigation shown, the power and precision in QTL identification are quite sensitive to the way the mean and covariance structures are modeled [14], [15], [19]. In reality, the true mean and covariance function are generally unknown. This raises a very practical issue in functional mapping. What we can do to improve mapping power and precision is by modeling the error distribution with more robust approaches such as the one proposed in this work. We expect that the method developed can enhance the full power of functional mapping in understanding the genetic architecture of dynamic traits.

## Supporting Information

Appendix S1 Derivation of the EM algorithm.
(PDF)
