# Supplementary material for: Functional Mapping of Dynamic Traits with Robust t-Distribution
Source: PLoS One. 2011 Sep 22;6(9):e24902. doi: 10.1371/journal.pone.0024902 (PMC3178556; doi:10.1371/journal.pone.0024902)
Supplement: Appendix S1 — Derivation of the EM algorithm. (PDF) [file pone.0024902.s001.pdf]

Appendix S1

Derivation of the EM algorithm

Define $c_i = 0$ or 1 if the QTL genotype is Qq or QQ, respectively. The p-dimensional random observations $\boldsymbol{y_i}$ $(i = 1...n)$ are generated independently from a two-component mixture of multivariate t distribution with proportions $\pi_{i|0}$ and $\pi_{i|1}$

$$f(\boldsymbol{y_i}; \Omega) = \sum_{j=0}^{1} \pi_{i|j} f_j(\boldsymbol{y_i}; \boldsymbol{\theta}_j) \tag{A1}$$

where $\pi_{i|j} = P(c_{i|j} = 1)$, $\Omega = (\boldsymbol{\theta}_0, \boldsymbol{\theta}_1)$ and $\boldsymbol{\theta}_j = (\boldsymbol{\mu}_j, \Sigma_j, \nu_j)$ $(j = 0, 1)$. Note that $\pi_{i|0} + \pi_{i|1} = 1$. The density $f_j(\boldsymbol{y_i}; \boldsymbol{\theta}_j)$ is defined in Eq. (1). By the property of the multivariate t distribution [26], n independent draws from $f_j(\boldsymbol{y_i}; \boldsymbol{\theta}_j)$ can be denoted as a weighted average of p-dimensional multivariate normal distributions with the weights τ_i following a Gamma distribution, i.e.

$$\boldsymbol{y_i}|\tau_i, c_{i|j} = 1 \sim N_p(\boldsymbol{\mu}_j, \Sigma_j/\tau_i) \quad \text{for} \quad i = 1, 2, ..., n \quad j = 0, 1$$

and

$$\tau_i|c_{i|j} = 1 \sim \text{Gamma}\left(\frac{\nu_j}{2}, \frac{\nu_j}{2}\right) \quad \text{independently for} \quad i = 1, 2, ..., n \quad \text{and} \quad j = 0, 1$$

where the Gamma(α,β) density function is defined as

$$\beta^{\alpha} \tau^{\alpha-1} \text{exp}(-\beta\tau)/\Gamma(\alpha) I_{(0,\infty)}(\tau), \quad (\alpha, \beta > 0)$$

The complete-data log-likelihood function can be expressed as

$$\ell^c(\boldsymbol{\Omega}) = \ell_1(\boldsymbol{\mu}, \boldsymbol{\Sigma}|\boldsymbol{y}, \boldsymbol{\tau}) + \ell_2(\boldsymbol{\nu}|\boldsymbol{\tau}) + \ell_3(\boldsymbol{\pi}) \tag{A2}$$

where

$$\ell_1(\boldsymbol{\mu}, \boldsymbol{\Sigma}|\boldsymbol{y}, \boldsymbol{\tau}) = \sum_{i=1}^{n}\sum_{j=0}^{1} c_{i|j}\{-\frac{1}{2}p\log(2\pi) - \frac{1}{2}\log|\Sigma_j| - \frac{1}{2}\tau_i(\boldsymbol{y_i} - \boldsymbol{\mu}_j)^{'}\Sigma_j^{-1}(\boldsymbol{y_i} - \boldsymbol{\mu}_j)\}$$

$$\ell_2(\boldsymbol{\nu}|\boldsymbol{\tau}) = \sum_{i=1}^{n}\sum_{j=0}^{1} c_{i|j}\{-\log\Gamma(\frac{\nu_j}{2}) + \frac{\nu_j}{2}\log\frac{\nu_j}{2} + \frac{\nu_j}{2}(\log\tau_i - \tau_i) - \log\tau_i\}$$

and

$$\ell_3(\boldsymbol{\pi}) = \sum_{i=1}^{n}\sum_{j=0}^{1} c_{i|j}\log(\pi_{i|j}),\ \boldsymbol{\pi} = (\pi_{i|0}, \pi_{i|1})^{'}$$

Then the MLEs of the parameters in $\boldsymbol{\Omega} = (\Omega_m, \Omega_c, \Omega_\nu)$ are obtained by solving

$$\frac{\partial}{\partial\Omega_s}\ell^c(\boldsymbol{\Omega}) = 0 \qquad \text{(A3)}$$

In this study, the two multivariate t components were assumed to have the same covariance structure and the same degree of freedom, i.e., $\Sigma_1 = \Sigma_2 = \Sigma$ and $\nu_1 = \nu_2 = \nu$. By choosing the uniform quadratic B-spline with degree 5, we obtained the normalized basis matrix $\mathbf{B}$ as

$$\mathbf{B} = \begin{bmatrix} 1 & 0 & 0 & 0 & 0 \\ 0.390625 & 0.5390625 & 0.0703125 & 0 & 0 \\ 0.0625 & 0.65625 & 0.28125 & 0 & 0 \\ 0 & 0.3828125 & 0.609375 & 0.0078125 & 0 \\ 0 & 0.125 & 0.75 & 0.125 & 0 \\ 0 & 0.0078125 & 0.609375 & 0.3828125 & 0 \\ 0 & 0 & 0.28125 & 0.65625 & 0.0625 \\ 0 & 0 & 0.0703125 & 0.5390625 & 0.390625 \\ 0 & 0 & 0 & 0 & 1 \end{bmatrix} \qquad \text{(A4)}$$

and $\boldsymbol{\xi_j}$ as the base genotypic vector for genotype j

$$\boldsymbol{\xi_j} = \begin{bmatrix} \xi_{0j} & \xi_{1j} & \xi_{2j} & \xi_{3j} & \xi_{4j} \end{bmatrix}^{'} \qquad \text{(A5)}$$

whose entries are the mean parameters to be estimated with the mean vector given by $\boldsymbol{\mu_j} = \mathbf{B}\boldsymbol{\xi_j}$.

For the SAD(1) covariance structure, it has very nice properties. For example, the inverse of the covariance matrix can be explicitly expressed (see [15] for more details).

Define

$$\mathbf{L} = \begin{bmatrix} 1 & 0 & 0 & \dots & \dots & 0 \\ -\phi & 1 & 0 & 0 & \dots & 0 \\ 0 & -\phi & 1 & 0 & \dots & 0 \\ \vdots & \dots & \dots & \dots & \dots & \vdots \\ 0 & \dots & 0 & -\phi & 1 & 0 \\ 0 & \dots & \dots & 0 & -\phi & 1 \end{bmatrix}$$

and

$$\boldsymbol{\Gamma}(\phi) = \begin{bmatrix} 1+\phi^2 & -\phi & 0 & \dots & 0 \\ -\phi & 1+\phi^2 & -\phi & \dots & 0 \\ 0 & -\phi & 1+\phi^2 & -\phi & \vdots \\ \vdots & \dots & \dots & \dots & 0 \\ 0 & \dots & -\phi & 1+\phi^2 & -\phi \\ 0 & \dots & 0 & -\phi & 1 \end{bmatrix}$$

The ML estimator of the unknown parameters can be obtained using the following EM algorithm. At the kth iteration in the **E-step**, the posterior probability of the observed trait vector $\boldsymbol{y_i}$ belonging to the genotype j can be expressed as

$$\hat{c}_{i|j}^{(k)} = \mathrm{E}(c_{i|j} = 1|\boldsymbol{y_i}; \hat{\Omega}^{(k)}) = \frac{\pi_{i|j} f_j(\boldsymbol{y_i}; \hat{\boldsymbol{\theta}}_j^{(k)})}{\pi_{i|0} f_0(\boldsymbol{y_i}; \hat{\boldsymbol{\theta}}_0^{(k)}) + \pi_{i|1} f_1(\boldsymbol{y_i}; \hat{\boldsymbol{\theta}}_1^{(k)})} \quad (j = 0, 1) \tag{A6}$$

where $\hat{\boldsymbol{\theta}}_j = (\hat{\boldsymbol{\mu}}_j, \hat{\sigma}^2, \hat{\phi}, \hat{\nu})$ $(j = 0, 1)$. And the conditional expectation of τ_i given $c_{i|j} = 1$ is calculated as

$$\hat{\tau}_{ij}^{(k)} = \mathrm{E}(\tau_i|\boldsymbol{y_i}, c_{i|j} = 1; \hat{\Omega}^{(k)}) = \frac{\hat{\nu}^{(k)} + p}{\hat{\nu}^{(k)} + (\boldsymbol{y_i} - \mathbf{B}\hat{\xi}_j^{(k)})' \frac{1}{\hat{\sigma^2}^{(k-1)}} \mathrm{L}'\mathrm{L}(\boldsymbol{y_i} - \mathbf{B}\hat{\xi}_j^{(k)})} \quad (j = 0, 1) \tag{A7}$$

In the **M-step**, the updates for ξ_j, σ^2 and ϕ are obtained as :

$$\hat{\xi}_j^{(k+1)} = \frac{\sum_{i=1}^{n} \hat{c}_{i|j}^{(k)} \hat{\tau}_{ij}^{(k)} \mathbf{B}'\mathrm{L}'\mathrm{L}\boldsymbol{y_i}}{\sum_{i=1}^{n} \hat{c}_{i|j}^{(k)} \hat{\tau}_{ij}^{(k)} \mathbf{B}'\mathrm{L}'\mathrm{L}} \tag{A8}$$

$$\widehat{\sigma^2}^{(k+1)} = \frac{\sum_{i=1}^{n} \sum_{j=0}^{1} \hat{c}_{i|j}^{(k)} \hat{\tau}_{ij}^{(k)} (\boldsymbol{y_i} - \mathbf{B}\hat{\xi}_j^{(k+1)})'\mathrm{L}'\mathrm{L}(\boldsymbol{y_i} - \mathbf{B}\hat{\xi}_j^{(k+1)})}{np} \tag{A9}$$

and

$$\hat{\phi}^{(k+1)} = \frac{\sum_{i=1}^{n} \sum_{j=0}^{1} \hat{c}_{i|j}^{(k)} \hat{\tau}_{ij}^{(k)} \sum_{k=1}^{p-1} [y_i(t_k) - \mathbf{B}'_k \hat{\xi}_j^{(k+1)}]'[y_i(t_{k+1}) - \mathbf{B}'_{k+1} \hat{\xi}_j^{(k+1)}]}{\sum_{i=1}^{n} \sum_{j=0}^{1} \hat{c}_{i|j}^{(k)} \hat{\tau}_{ij}^{(k)} \sum_{k=1}^{p-1} [y_i(t_k) - \mathbf{B}'_k \hat{\xi}_j^{(k+1)}]^2} \tag{A10}$$

Given the degree of freedom of the multivariate t distribution, the above MLEs will be updated in closed form. To update ν, we obtain $\hat{\nu}^{(k+1)}$ by finding the solution to the equation:

$$\begin{aligned} \textstyle\sum_{i=1}^{n} \sum_{j=0}^{1} \hat{c}_{i|j}^{(k)} \{ \quad & -\psi(\tfrac{\nu}{2}) + \log(\tfrac{\nu}{2}) + 1 + \log(\hat{\tau}_{ij}^{(k)}) - \hat{\tau}_{ij}^{(k)} \\ & +\psi(\tfrac{\hat{\nu}^{(k)}+p}{2}) - \log(\tfrac{\hat{\nu}^{(k)}+p}{2}) \} = 0 \qquad (j = 0, 1) \end{aligned} \tag{A11}$$

where the digamma function $\psi(x)$ is defined as $\psi(x) \equiv \frac{d(log\Gamma(a))}{da} = \frac{\Gamma(a)}{\Gamma'(a)}$. The one-dimensional search for $\hat{\nu}^{(k+1)}$ is time consuming. Shoham [27] provided a direct approximation solution of accurateness $|\nu - \nu^*| < 10^{-3}$ to this nonlinear equation, i.e.

$$\nu^* = \frac{2}{h + \log h - 1} + 0.0416(1 + \mathrm{erf}(0.6594 \times \log(\frac{2.1971}{h + \log h - 1}))) \tag{A12}$$

where

$$h = -\frac{1}{n} \sum_{i=1}^{n} \sum_{j=0}^{1} \hat{c}_{i|j}^{(k)} \{\log(\hat{\tau}_{ij}^{(k)}) - \hat{\tau}_{ij}^{(k)} + \psi(\frac{\hat{\nu}^{(k)} + p}{2}) - \log(\frac{\hat{\nu}^{(k)} + p}{2})\}$$

The above procedures are iterated until certain convergence criterion is achieved. The converged values are the MLEs of the parameters. Note that in the above EM algorithm, we used a grid search method to estimate the QTL location instead of estimating the QTL segregation parameters in Ω_l directly.
